# The Biogenesis of Dengue Virus Replication Organelles Requires the ATPase Activity of Valosin-Containing Protein

**DOI:** 10.3390/v13102092

**Published:** 2021-10-18

**Authors:** Clément Mazeaud, Anaïs Anton, Felix Pahmeier, Aïssatou Aïcha Sow, Berati Cerikan, Wesley Freppel, Mirko Cortese, Ralf Bartenschlager, Laurent Chatel-Chaix

**Affiliations:** 1 Centre Armand-Frappier Santé Biotechnologie, Institut National de la Recherche Scientifique, Laval, QC H7V 1B7, Canada; Clement.Mazeaud@inrs.ca (C.M.); Anais.Anton@inrs.ca (A.A.); Aicha.Sow@inrs.ca (A.A.S.); wesley.freppel@inrs.ca (W.F.); 2 Center for Integrative Infectious Disease Research (CIID), Department of Infectious Diseases, Molecular Virology, Heidelberg University, D-69120 Heidelberg, Germany; felix.pahmeier@googlemail.com (F.P.); Berati.Cerikan@med.uni-heidelberg.de (B.C.); m.cortese@tigem.it (M.C.); Ralf.Bartenschlager@med.uni-heidelberg.de (R.B.); 3 German Center for Infection Research (DZIF), Heidelberg Partner Site, D-69120 Heidelberg, Germany; 4 Center of Excellence in Research on Orphan Diseases-Fondation Courtois, Montreal, QC H7V 1B7, Canada; 5 Réseau Intersectoriel de Recherche en Santé de l’Université du Québec, Québec, QC H7V 1B7, Canada

**Keywords:** dengue virus, valosin-containing protein, NS4B, viral replication organelles, endoplasmic reticulum

## Abstract

The dengue virus (DENV) causes the most prevalent arthropod-borne viral disease worldwide. While its incidence is increasing in many countries, there is no approved antiviral therapy currently available. In infected cells, the DENV induces extensive morphological alterations of the endoplasmic reticulum (ER) to generate viral replication organelles (vRO), which include convoluted membranes (CM) and vesicle packets (VP) hosting viral RNA replication. The viral non-structural protein NS4B localizes to vROs and is absolutely required for viral replication through poorly defined mechanisms, which might involve cellular protein partners. Previous interactomic studies identified the ATPase valosin-containing protein (VCP) as a DENV NS4B-interacting host factor in infected cells. Using both pharmacological and dominant-negative inhibition approaches, we show, in this study, that VCP ATPase activity is required for efficient DENV replication. VCP associates with NS4B when expressed in the absence of other viral proteins while in infected cells, both proteins colocalize within large DENV-induced cytoplasmic structures previously demonstrated to be CMs. Consistently, VCP inhibition dramatically reduces the abundance of DENV CMs in infected cells. Most importantly, using a recently reported replication-independent plasmid-based vRO induction system, we show that *de novo* VP biogenesis is dependent on VCP ATPase activity. Overall, our data demonstrate that VCP ATPase activity is required for vRO morphogenesis and/or stability. Considering that VCP was shown to be required for the replication of other flaviviruses, our results argue that VCP is a pan-flaviviral host dependency factor. Given that new generation VCP-targeting drugs are currently evaluated in clinical trials for cancer treatment, VCP may constitute an attractive broad-spectrum antiviral target in drug repurposing approaches.

## 1. Introduction

The dengue virus (DENV) causes the most prevalent arthropod-borne viral disease worldwide with approximately 100 million symptomatic infections annually. The DENV is endemic in 100 countries and its incidence has increased over 30-fold in the last 50 years. The DENV is transmitted to humans through the bite of *Aedes Aegypti* or *Aedes albopictus* female mosquitos. Infections can induce symptoms with a broad spectrum of severity, which include hemorrhagic fever, shock syndrome, and death in some cases [1,2,3]. While the safety of the only approved dengue vaccine in seronegative individuals has been questioned [4], there are no antivirals currently available.

DENV is an enveloped positive single-stranded RNA virus that belongs to the genus *Flavivirus* within the family *Flaviviridae*. Following virus entry into the cell, the viral RNA genome (vRNA) is released in the cytosol and translated into a single large polyprotein, which is cleaved by host and viral proteases to generate 10 mature viral proteins. On one hand, structural proteins capsid (C), pre-membrane (prM), and envelope (E), together with vRNA, drive the assembly of viral particles. On the other hand, nonstructural proteins NS1, NS2A, NS2B, NS3, NS4A, NS4B, and NS5 are responsible for intracellular vRNA replication, a process which is believed to occur within endoplasmic reticulum (ER)-derived ultrastructures, called vesicle packets (VP) [5,6]. These ~90 nm-wide spherules are induced through the invagination of the ER, pack in plane in this organelle, and contain most (if not all) viral non-structural proteins, such as NS3, NS4B, and NS5, as well as double-stranded RNA (dsRNA), the vRNA replication intermediate [7,8]. In addition, the DENV induces the tight accumulation of smooth ER membranes, termed convoluted membranes (CM), which are structurally related to cubic membranes. These dsRNA-free ultrastructures are presumably not directly involved in the vRNA synthesis process. Since they are connected to VPs within the same ER network and are enriched in NS3 protease [8,9,10], it was proposed that they are involved in co-translational polyprotein maturation and VP biogenesis. However, it was never firmly demonstrated. More recently, their morphogenesis was functionally linked to the viral subversion of cellular processes, which are potentially detrimental to viral replication, such as innate immunity and apoptosis [9,11]. Finally, assembled virions accumulate in regular arrays within ER-derived enlarged cisternae [7,8]. These viral replication organelles (vROs) (or viral replication factories), generated through the extensive remodeling of the ER, constitute a cytoplasmic compartment that is favorable to vRNA amplification. Such virus-induced structures are also observed in cells infected with other flaviviruses, such as Zika virus (ZIKV), West Nile virus (WNV), Japanese encephalitis virus (JEV), and tick-borne encephalitis virus (TBEV) [10,12,13,14,15,16]. This strongly suggests that the molecular mechanisms governing vRO morphogenesis are conserved across the *Flavivirus* genus. However, the host and viral determinants of vRO biogenesis remain poorly defined. NS4B, an integral membrane protein, was proposed to be important for this process, since it is enriched in VPs and CMs, and is absolutely required for RNA replication [8,9,17,18,19,20]. Interestingly, NS4B was shown to be the viral target of several potent DENV inhibitors [21,22,23,24,25], illustrating the importance of this viral protein in the life cycle. Nevertheless, NS4B precise functions are poorly understood, while they may partly involve interactions with specific host factors. 

Interactome studies have identified the AAA+ ATPase valosin-containing protein (VCP, also called p97) as a DENV NS4B protein partner, although such interaction was never validated [9,26]. We have recently reported that VCP also associates with ZIKV NS4B [11]. VCP is a ubiquitous protein with multiple roles related to proteostasis. Notably, it contributes to ER-associated degradation and mitochondria-associated degradation by retrotranslocating misfolded membrane proteins and targeting these substrates to the proteasome. It can also disassemble protein aggregates [27,28,29,30,31,32,33,34]. At the clinical level, multiple familial missense mutations are associated to severe genetic neurological diseases, such as frontotemporal dementia, classical amyotrophic lateral sclerosis, inclusion body myopathy, and Paget’s disease of bone, alone or in combination [35,36,37,38]. Importantly, the inhibition of VCP ATPase activity impairs the replication of ZIKV, WNV, JEV, and yellow fever virus (YFV) in cell culture [11,39,40,41,42,43], demonstrating that this host factor regulates flavivirus replication, possibly through a conserved mechanism. In ZIKV-infected cells, we have shown that VCP and NS4B mostly colocalize in large structures, which presumably are the CMs [11]. Very interestingly, a four-hour treatment of ZIKV-infected cells with the selective VCP ATPase inhibitors CB-5083 or NMS-873 drastically reduced the abundance and size of the CMs, demonstrating that VCP is required for CM stability. This alteration correlated with an increase of ZIKV-induced apoptosis, supporting that CMs regulate cellular processes. Whether this specific VCP function is conserved for all flaviviruses, including the DENV, is unknown. In such treatment conditions, ZIKV VPs could not be observed. While we could not quantify this phenotype and exclude that it was due to an indirect consequence of vRNA synthesis shutdown, this raised the hypothesis that VCP also regulates VP morphogenesis.

Using pharmacological and genetic inhibition approaches, we show in this study that VCP ATPase regulates DENV replication, similarly to other flaviviruses. VCP associates with NS4B when expressed alone or in infected cells, and these proteins colocalize within large DENV-induced cytoplasmic structures previously demonstrated to be CMs [9]. Consistently, VCP inhibition dramatically reduces the abundance of DENV CMs. Most importantly, using a recently reported replication-independent plasmid-based vRO induction system [44,45], we further show that *de novo* VP biogenesis requires VCP ATPase activity. This study supports that VCP is a pan-flaviviral host dependency factor and constitutes an attractive antiviral target, especially considering that new generation VCP drugs are currently evaluated in clinical trials for cancer treatment. 

## 2. Materials and Methods

### 2.1. Cells, DNAs, Viruses, and Reagents

293T, HeLa, VeroE6 and Huh7.5 cells (kind gifts from Drs. Frédérick-Antoine Mallette, Tom Hobman, Anil Kumar, and Patrick Labonté) were cultured in a DMEM (Thermo-Fisher) supplemented with 10% fetal bovine serum (FBS; Wisent Inc., Saint-Jean-Baptiste, QC, Canada cat#098150), 1% penicillin-streptomycin (PS; Thermo-Fisher), and 1% non-essential amino acids (NEAA; Thermo-Fisher). The generation of Huh7.5-T7 and Huh7-Lunet-T7, which stably express the T7 RNA polymerase, was previously reported [11,44,45]. Huh7.5-T7 and Huh7-Lunet-T7 cells were cultured in DMEM/10% FBS/1% PS/1% NEAA, in the presence of 5 µg/mL blasticidin (Thermo-Fisher, Waltham, MA, USA) and 5 µg/mL zeocin (Invitrogen, Waltham, MA, USA), respectively. 

The cloning of DENV2 16681s molecular clones (pFK-DVs and pFK-DVs-R2A), NS4B-encoding pTM constructs, pTM/DENV Δ5’ SLAB-3’ wt-Ribozyme (pIRO-D), and VCP-expressing lentiviral pWPI plasmids was previously reported [11,17,44,46]. 

Wild-type DENV2 16681s and Rluc-expressing reporter viruses were generated in VeroE6 cells following electroporation of in vitro transcribed RNA genomes. Briefly, following the linearization of pFK-DVs or pFK-DVs-R2A plasmids, in vitro transcription was performed using the mMESSAGE mMACHINE kit (Thermo-Fisher) with the SP6 RNA polymerase. RNAs were purified according to the manufacturer’s instructions. Next, sub-confluent VeroE6 cells were detached by trypsinization and collected in complete DMEM. The cells were washed once in PBS and resuspended in a cytomix buffer (120 mM KCl, 0.15 mM CaCl_2_, 10 mM potassium phosphate buffer (pH 7.6), 25 mM HEPES (pH 7.6), 2 mM EGTA, 5 mM MgCl_2_ (pH 7.6) freshly supplemented with 2 mM ATP, and 5 mM glutathione) at a density of 1.5 × 10^7^ cells/mL. A total of 10 µg of in vitro-transcribed RNA were mixed with 400 µL of the cell suspension, transferred to an electroporation cuvette (0.4 cm gap width; Bio-Rad, Hercules, CA, USA), and pulsed once with a Gene Pulser Xcell Total System (Bio-Rad) at 975 µF and 270 V, typically resulting in time constants between 18 and 20 ms. Immediately after pulsing, the cells were transferred to pre-warmed complete DMEM and seeded in a 15 cm dish. On the next day, the cell culture medium was replaced with complete DMEM. Virus-containing cell culture supernatants were harvested once a day at 4, 5, 6, and 7 days post-electroporation, filtered through a 0.45 µm syringe filter, and supplemented with 10 mM HEPES (pH 7.5). DENV1 Hawaii, DENV2 New Guinea C, DENV3 H87, and DENV4 H241 were all generously provided by Professor Tom Hobman (University of Alberta) and amplified in VeroE6 cells for 4 to 7 days. Virus aliquots were stored at −80 °C until use. Infectious titers were determined by plaque assays. 

NMS-873 and CB-5083 were obtained from Millipore-Sigma (Burlington, MA, USA) and Selleck Chemicals (Houston, TX, USA), respectively. Mouse monoclonal anti-VCP (ab11433) was purchased from Abcam (Cambridge, United Kingdom). Rabbit anti-DENV NS4B (GTX124250) and mouse monoclonal anti-DENV NS3 (GTX629477) were obtained from Genetex (Irvine, CA, USA). The generation of rat polyclonal anti-DENV NS3 antibodies by Medimabs (Montréal, QC, Canada) was previously described [11]. 

### 2.2. Lentiviral Transduction

Lentiviruses encoding wild type or dominant negative HA-tagged VCP proteins were produced in 293T cells and titrated in HeLa cells exactly as previously described [11,45]. Huh7.5 cells were transduced with lentiviruses at a MOI of 1 in the presence of 8 µg/mL polybrene. A DENV infection was carried out 2 days post-transduction and viability, luciferase, and plaque assays were performed 4 days post-transduction.

### 2.3. Cell Viability Assays

For NMS-873 and CB-5083 CC_50_ values determination, cell viability was evaluated using the CellTiter-Glo Luminescent Cell Viability Assay kit (Promega, Madison, WI, USA) according to the manufacturer’s instructions. Luminescence was measured with a Spark^®^ multimode microplate reader (Tecan, Charlotte, NC, USA). Viability of transduced cells was assessed using MTT assays exactly as previously described [11].

### 2.4. Plaque Assays

200,000 VeroE6 cells were seeded in 24-well plates. Twenty-four hours later, cells were infected in duplicates with 200 µL virus samples that were subjected to 10-fold serial dilutions in a complete DMEM (10^1^ to 10^6^-fold dilution). Two hours post-infection, the inoculum was removed and a serum-free MEM (Life Technologies, Waltham, MA, USA) containing 1.5% carboxymethylcellulose (Millipore-Sigma, Burlington, MA, USA) was added. After 4 to 7 days, depending on the DENV strain, cells were fixed in 5% formaldehyde. Following washes with water, cells were stained with 1% crystal violet/10% ethanol for 15–30 min and then washed again with water. Plaques were counted and infectious titers were determined. The detection limit of this assay is 25 PFU/mL.

### 2.5. Luciferase Assays

Cells that were infected with the reporter virus DVs-R2A were lysed in 100–200 µL of lysis buffer (0.1% Triton X-100, 25 mM glycylglycine (pH 7.8), 15 mM MgSO4, 4 mM EGTA (pH 8), and 1 mM DTT). A total of 150 μL of assay buffer (25 mM glycylglycine (pH 7.8), 15 mM MgSO_4_, 4 mM EGTA (pH 8), 15 mM K_2_PO_4_ (pH 7.8), and 1.43 µM benzyl-coelenterazine (Prolume, Pinetop-Lakeside, AZ, USA) were injected to 30 μL of the lysate. Five seconds after this injection, the luminescence was measured for one second with a Spark^®^ multimode microplate reader (Tecan).

### 2.6. Transfection

All cell transfections with NS4B-expressing pTM plasmids were carried out with the TransIT-LT1 Transfection Reagent (Mirus, Madison, WI, USA) according to the manufacturer’s instructions and exactly as previously described [11,45]. Cells were subjected to co-immunoprecipitation assays or immuno-labelling for confocal microscopy 16 h post-transfection.

### 2.7. Immunofluorescence-Based Confocal Microscopy

Transfected or infected cells were grown on glass coverslips, washed twice with phosphate-buffered saline (PBS), fixed with 4% paraformaldehyde in PBS, and permeabilized with PBS-0.2% Triton X-100 for 15 min. Cells were blocked during 1 h with PBS containing 5% bovine serum albumin (BSA) and 10% goat serum (Thermo-Fisher), and then incubated with primary antibodies for 2 h at room temperature in the dark. Following three washes in PBS, the coverslips were incubated with Alexa Fluor-conjugated secondary antibodies (Life Technologies, Waltham, MA, USA) for 1 h at room temperature in the dark. The coverslips were then washed three times with PBS during 10 min, and the nuclei were stained with 4′, 6′-diamidino-2-phenylindole (DAPI; Life Technologies). Following three final washes with PBS and one with water, the coverslips were mounted on slides with Fluoromount-G (Southern Biotechnology Associates, Birmingham, AL, USA). The cells were imaged with a LSM780 confocal microscope (Carl Zeiss Microimaging, Oberkochen, Germany) at the Confocal Microscopy Core Facility of the INRS-Centre Armand-Frappier Santé Biotechnologie.

### 2.8. Co-Immunoprecipitation Assays

Transfected cells were washed twice in PBS and incubated during 20 min on ice in a lysis buffer (0.5% Dodecyl-B-D-maltoside, 100 mM NaCl, 20 mM Tris (pH 7.5), 50 mM NaF, and EDTA-free protease inhibitors (Roche, Mannheim, Germany). Lysed cells were centrifuged during 15 min at 13,000 rpm at 4 °C, and supernatants were collected. Resulting cell extracts were incubated with 50 µL of a 50/50 slurry of mouse monoclonal anti-HA coupled to agarose beads (Millipore-Sigma) at 4 °C. Three hours later, the resin was washed twice with lysis buffer and twice with a solution containing 150 mM NaCl and 50 mM Tris (pH 7.5). Resin-associated proteins were collected by a first elution with PBS-5% SDS, and by a second one with PBS. The eluates were pooled and precipitated overnight at −20 °C by adding 4 volumes of acetone. The precipitated proteins were centrifuged during 1 h at 13,000 rpm. The resulting pellets were air-dried, resuspended in loading buffer, and subjected to Western blot analysis.

### 2.9. Transmission Electron Microscopy

Huh7.5 were grown on Lab-tech^®^ chamber SlideTM (Thermo Fisher) and infected with DENV2 16681s at a MOI of 1. Two days post-infection, cells were treated with either 0.2% DMSO, 20 µM CB-5083, or 20 µM NMS-873 for 4 h. Following three washes with PBS, cells were fixed overnight at 4 °C in 2.5% glutaraldehyde in 0.1 M sodium cacodylate buffer (pH 7.4). Specimens were treated with osmium, dehydrated, and Epon-resin embedded at the McGill University Facility for Electron Microscopy Research exactly as previously described [11]. Ultrathin serial sections (90–100 nm thick) were prepared with a Diatome diamond knife using a Leica Microsystems EM UC7 ultramicrotome and transferred onto 200-mesh copper grids. The grids were stained with 4% uranyl acetate for 6 min and Reynold’s lead for 5 min. Samples were imaged with a FEI Tecnai G2 Spirit 120 kV TEM equipped with a Gatan Ultrascan 4000 CCD Camera Model 895 (Gatan, Pleasanton, CA) located at the McGill University Facility for Electron Microscopy Research. The DENV CM analysis was performed using Fiji software.

For the replication-independent VP induction, Huh7-Lunet-T7 cells were transfected with pTM/DENV Δ5’ SLAB-3’ wt-Ribozyme (pIRO-D) plasmid exactly as previously described [44,45]. Four hours later, cells were treated with DMSO, 2.5 µM CB-5083 or 1 µM NMS-873 for 12 h. Sixteen hours post-transfection, the cells were fixed with an EM fixative (50 mM sodium cacodylate buffer, 50 mM KCl, 2.6 mM MgCl_2_, 2.6 mM CaCl_2_, 1% paraformaldehyde, 2.5% glutaraldehyde, and 2% sucrose (pH 7.4)) for 30 min at room temperature. In parallel to the EM samples, transfection efficiency was evaluated by immunofluorescence analysis as described above. After the fixation of the EM samples, they were washed 5 times with 50 mM sodium cacodylate and incubated with 2% osmium tetroxide in 50 mM cacodylate buffer for 40 min on ice. Following 3 additional washes with EM-grade water, samples were incubated for 30 min with 0.5% uranyl acetate in water, rinsed three times with water, and subject to dehydration in a graded ethanol series (from 40% to 100%). Samples were embedded in epoxy resin and the polymerized for 48 h at 60 °C. Ultrathin sections of 70 nm were produced by sectioning with a UC6 ultramicrotome (Leica Microsystems, Wetzlar, Germany) and sections were collected, counterstained with uranyl acetate and lead citrate, and examined with a JEOL JEM-1400 transmission electron microscope. For the VP measurements, the EM images were analyzed with Fiji software and the number and diameter of the VPs were measured. At least 15 cells were counted for each condition in 2 experiments.

## 3. Statistical Analysis

**Figure 1:**
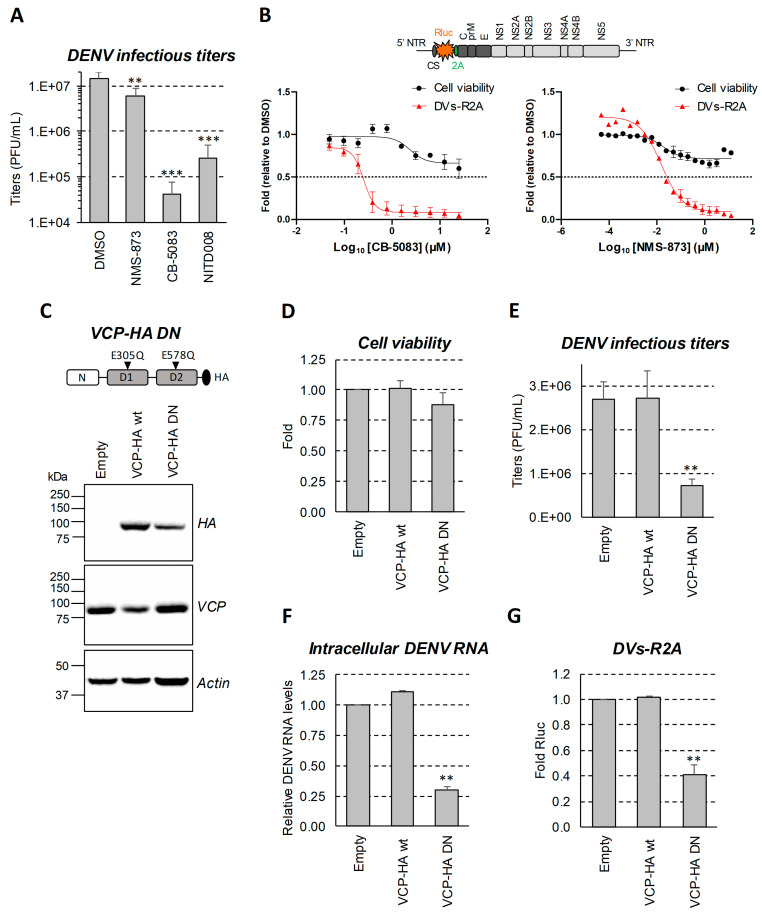
VCP ATPase inhibition impairs DENV replication. (**A**) Huh7.5 cells were infected with DENV2 16681s at a MOI of 0.05. The day after, the cells were treated with DMSO, 50 nM NMS-873, 0.5 µM CB-5083, or 10 µM NITD008 as positive control. After 24 h (2 days post-infection), cell supernatants were collected, and plaque assays were performed. (**B**) Huh7.5 cells were infected with the DENV2 16681s Renilla luciferase (Rluc)-expressing reporter virus (DVs-R2A) at a MOI of 0.001. One day post-infection, the infected cells were treated with various concentration of CB-5083 or NMS-873. After 24 h (2 days post-infection), Rluc assays were performed. Cell viability was measured in uninfected cells by CellTiter-Glo luminescent assays. The plotted data are relative to the DMSO treatment control. The dot line is a marker of a 50% decrease in viability or replication. NTR: non-translated region; 5′ CS: 5′ cyclization sequence; 2A: *Tosea asigna* virus 2A cleavage site at the C-terminus of Rluc to ensure proper processing after polyprotein synthesis. (**C**,**D**) Huh7.5 cells were transduced with lentiviruses expressing wild-type VCP or the depicted dominant negative VCP E305Q/E578Q mutant (MOI = 1). Four days post-transduction, the cells were collected and analyzed by Western blotting using the indicated antibodies (**C**) or subjected to MTT assays (**D**). (**E**,**F**) The cells were transduced as in (**C**). Two days post-transduction, the cells were infected with DENV2 16681s at a MOI of 0.05. Two days post-infection (four days post-transduction), extracellular infectious titers and relative intracellular DENV RNA levels were determined using plaque assays (**E**) and RT-qPCR (**F**), respectively. (**G**) Cells were transduced as in (**C**). Two days post-transduction, cells were infected with DVs-R2A at a MOI of 0.001. Two days post-infection (4 days post-transduction), viral replication was measured using Rluc assays. All results are representative of two or three independent experiments. **: *p*-value ≤ 0.01; ***: *p*-value ≤ 0.001.

All Student t-tests were unpaired and two-tailed. In Figure 1A,E and Supplementary Materials Figure S1, because of variations in the reference titer absolute values (DMSO or Empty pWPI conditions) between independent experiments, t-tests challenging the significance of the phenotypes were performed with normalized values reflecting the percentage of replication as compared to the control.

## 4. Results

### 4.1. VCP ATPase Activity Is Required for Efficient DENV Replication 

To investigate whether VCP ATPase is required for DENV replication, Huh7.5 hepatocarcinoma cells were infected with the DENV (serotype 2, strain 16681s) at a multiplicity of infection (MOI) of 0.05, and treated for 24 h with 2 selective VCP ATPase inhibitors, namely CB-5083 or NMS-873, which exhibit 2 different modes of action (ATP-competitive vs. allosteric non-ATP-competitive, respectively) [47,48,49,50]. At concentrations previously shown to be non-toxic in this cell line [11], both treatments significantly reduced the production of infectious viruses (Figure 1A). Remarkably, DENV infectious titers were reduced by more than 100-fold following CB-5083 treatment at 0.5 µM. NMS-873 treatment reduced virus production by 60%. At this low MOI, the infection did not induce additional cell death (data not shown). Using a reporter DENV virus expressing the Renilla luciferase (Rluc) in frame with the polyprotein [46] and thus, allowing to indirectly measure intracellular viral replication in luciferase assays, we determined that the half maximal effective concentration (EC_50_) of CB-5083 and NMS-873 were 0.25 µM and 0.02 µM, respectively (Figure 1B). In contrast, their 50% cytotoxic concentration CC_50_ were above 25 µM. 

In order to confirm that DENV replication requires VCP ATPase activity, we expressed in Huh7.5 cells the VCP dominant-negative E305Q/E578Q mutant (VCP-HA DN) through lentiviral transduction (Figure 1C). In this set-up, VCP-HA DN was produced at sub-endogenous levels since the total VCP amounts (detected with anti-VCP antibodies) were not increased. This mutant was shown to impair the enzymatic activity of VCP hexamers [51]. In contrast to cells expressing HA-tagged wild-type VCP, virus production and vRNA intracellular levels were decreased when cells expressed VCP-HA-DN, while cell viability was not impacted (Figure 1D–F). The same phenotype was observed with DENV reporter virus using luciferase activity as a read-out of viral replication (Figure 1G).

Finally, we have confirmed the role of VCP in the DENV life cycle by extending our analysis to another serotype 2 strain, namely the DENV2 New Guinea C, as well as to DENV strains belonging to serotypes 1, 3, and 4. As shown in Supplementary Materials Figure S1, a 24 h treatment of infected cells with CB-5083 drastically decreased viral production in all cases. Overall, these results show that VCP ATPase activity is required for efficient DENV replication.

### 4.2. VCP Associates with DENV NS4B Independently of Other Viral Proteins 

**Figure 2:**
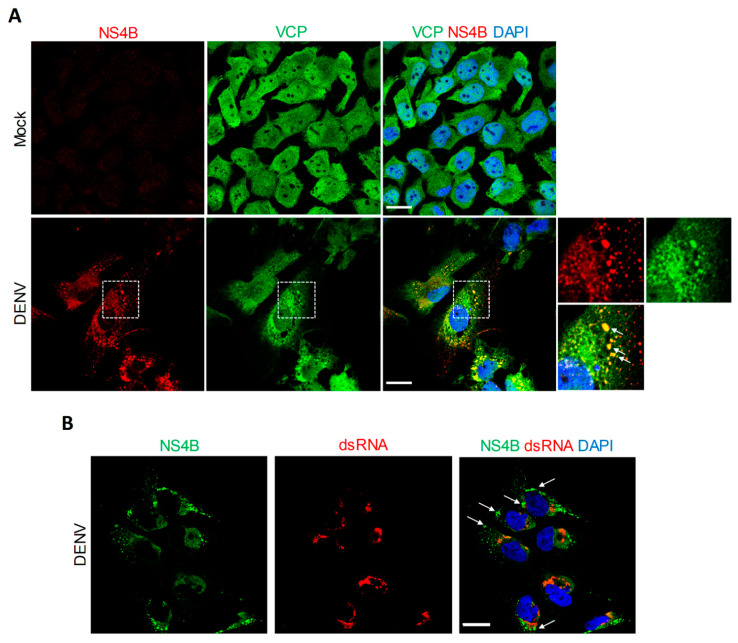
VCP associates with the DENV NS4B in infected cells. (**A**) Huh7.5 cells were infected with DENV2 16681s (MOI = 1) or left uninfected. Two days later, cells were fixed, immunolabeled with anti-VCP and anti-NS4B antibodies, and imaged by confocal microscopy. The bottom right panels show single channel and merged images of the magnified area indicated with the dashed square (~2.4-fold magnification). (**B**) Huh7.5 were prepared exactly as in (**A**) and labeled with anti-NS4B and anti-dsRNA antibodies. White arrows indicate colocalization foci.

**Figure 3:**
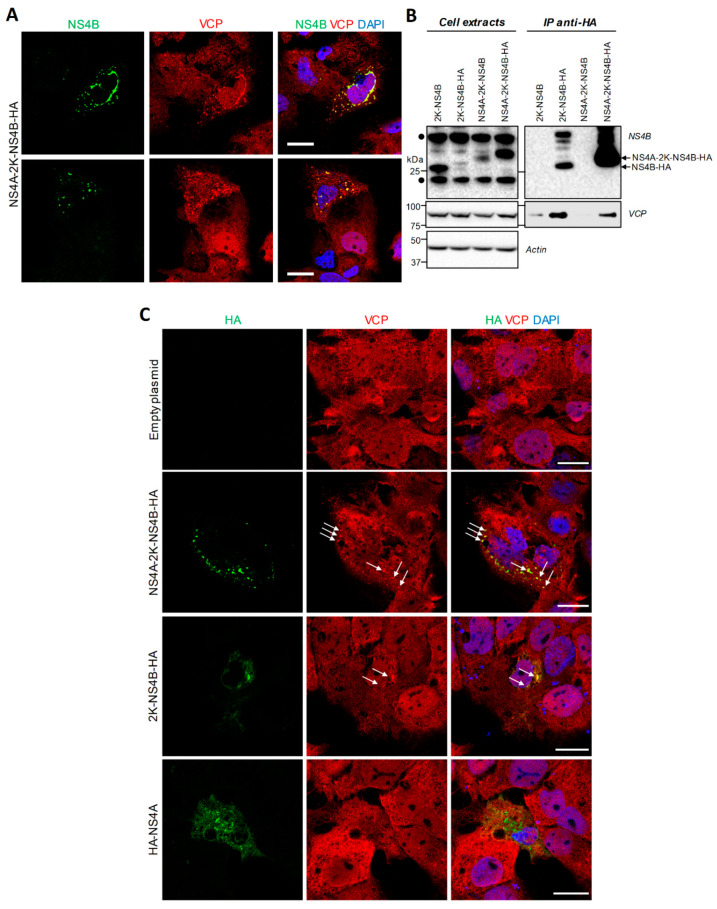
VCP associates with DENV NS4B when expressed alone as a precursor or a mature protein. (**A**) Huh7.5-T7 cells were transfected with a plasmid expressing DENV2 16681s NS4A-2K-NS4B-HA. Sixteen hours post-transfection, the cells were fixed, immunolabeled with anti-VCP and anti-NS4B antibodies, and imaged by confocal microscopy. Scale bar: 20 µm. (**B**) Huh7.5-T7 cells were transfected with the plasmids expressing the indicated viral proteins. Sixteen hours post-transfection, cells extracts were prepared and subjected to co-immunoprecipitation directed against HA. Resulting eluates and cell extracts were analyzed by Western blotting using the indicated antibodies. The DENV NS4A-2K-NS4B typically exhibits a lower molecular weight than expected because of a faster in-gel migration. The black dots indicate non-specific signals generated by the anti-NS4B antibodies. (**C**) Huh7.5-T7 cells were transfected with plasmids expressing the indicated DENV proteins. Sixteen hours post-transfection, the cells were fixed, immunolabeled with anti-VCP and anti-HA antibodies, and imaged by confocal microscopy. White arrows indicate colocalization foci. Scale bar: 20 µm.

Our previous interactomic study has identified VCP as a protein partner of NS4B in infected cells [9]. To obtain more insight about a potential co-opting mechanism of VCP by the DENV through NS4B, VCP localization in DENV-infected Huh7.5 cells was analyzed using confocal microscopy. In uninfected conditions, VCP did exhibit a diffuse distribution throughout the cell. In contrast, two days post-infection, VCP distribution was drastically altered (Figure 2A). Indeed, this protein accumulated in large NS4B-enriched structures (Figure 2A, white arrows), which we have previously demonstrated to be devoid of dsRNA and to be CMs using correlative light-electron microscopy (CLEM) (Figure 2B, white arrows) [9]. In addition, VCP relocalized to the NS4B-positive perinuclear area (Figure 2A) in which dsRNA (i.e., replication complexes) typically accumulates (Figure 2B) [9]. Although these phenotypes are consistent with the reported NS4B/VCP interaction in infected cells [9], we next assessed whether it required the contribution of other viral proteins. We transfected Huh7.5 cells stably expressing the T7 RNA polymerase (Huh7.5-T7) with a plasmid encoding NS4B as the HA-tagged NS4A-2K-NS4B precursor under the control of the T7 promoter, exactly as reported before [9,17]. We initially chose to express this precursor because it is readily detectable by confocal microscopy, with a much punctated distribution as compared to the one of the pseudo-mature 2K-NS4B [9,17]. Indeed, in transfected cells, NS4B localized in large punctae in which VCP partially redistributed (Figure 3A,C, white arrows). Although such punctae were less abundant in 2K-NS4B-HA-expressing cells, they were also enriched in VCP (Figure 3C, white arrows). In contrast, VCP distribution in HA-NS4A-expressing cells was comparable to that in control cells. This suggests that NS4B and VCP can interact in the absence of other proteins. To confirm this, we performed co-immunoprecipitations directed against the HA-tag with cells expressing HA-tagged NS4B proteins (Figure 3B). Endogenous VCP was readily detected in the eluates when immature or mature NS4B-HA was pulled-down, whereas very little, if any, was present in control conditions in which untagged NS4B proteins were expressed. These results demonstrate that VCP interaction with DENV NS4B does not absolutely require other viral proteins.

### 4.3. The Abundance of DENV Convoluted Membranes in Infected Cells Depends on VCP ATPase Activity 

**Figure 4:**
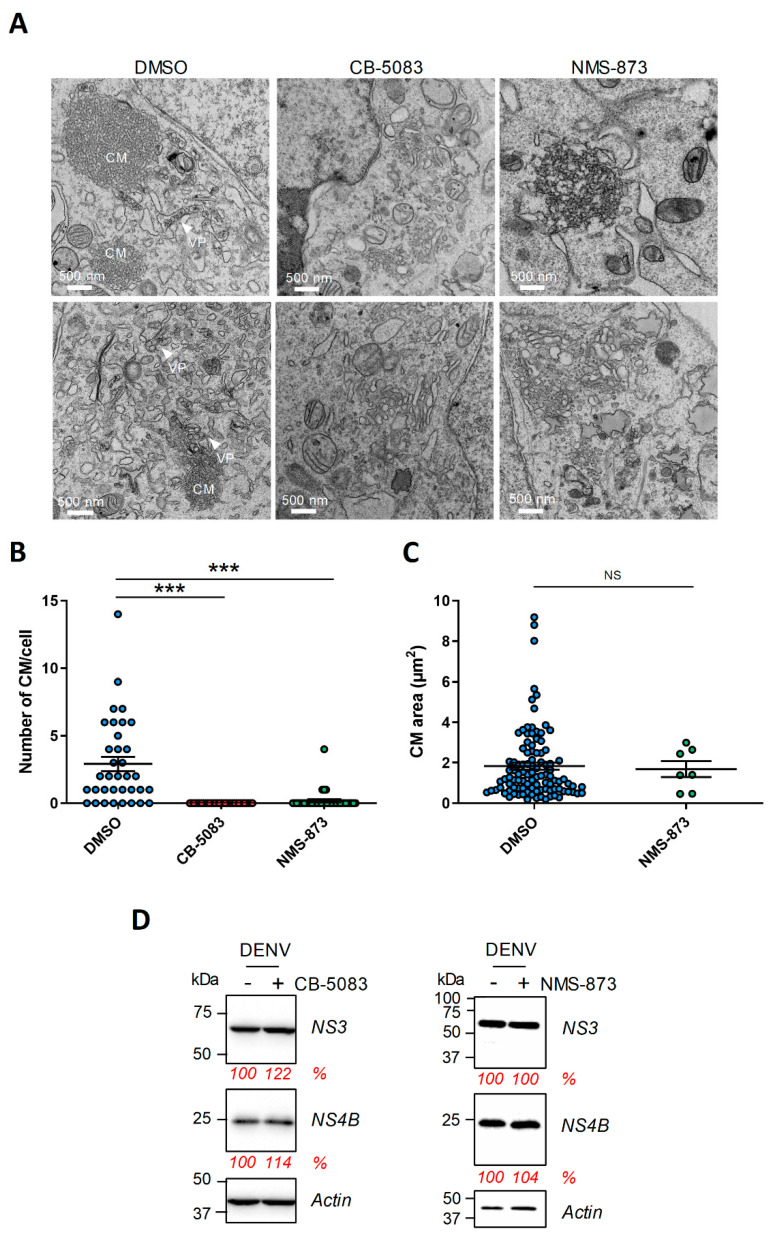
VCP ATPase activity is required for the stability of the DENV convoluted membranes. (**A**) Huh7.5 cells were infected with DENV2 16681s at a MOI of 1. Two days post-infection, cells were treated with DMSO, 20 µM CB-5083, or 20 µM NMS-873. After a 4 h treatment, the cells were prepared for imaging by transmission electron microscopy. CM: convoluted membranes; VP: vesicle packets. (**B**,**C**) More than 33 cells from each condition were analyzed for CM abundance (**B**) and size (**C**). ***: *p*-value ≤ 0.001; NS: not significant. (**D**) Huh7.5 cells were infected with DENV2 16681s at a MOI of 1. Two days post-transfection, cells were treated with DMSO, 20 µM CB-5083 for four hours, or 20 µM NMS-873 for one hour. Cell extracts were prepared and analyzed by Western blotting with the indicated antibodies. The relative abundance of NS3 and NS4B (shown in red) was quantified after normalization to actin levels using the ImageLab software (Bio-Rad).

Since VCP accumulates in large structures that are reminiscent of CMs in DENV-infected cells, we hypothesized that its ATPase activity is required for their stability and/or biogenesis. To test this, at 2 days post-infection, the cells were treated with high concentrations of NMS-873 or CB-5083 for 4 h and then prepared for CM imaging using transmission electron microscopy. In parallel, we controlled that the infection efficiency was 90–100% at this time post-infection and MOI. In control DMSO-treated infected cells, CMs as well as VPs were readily observed (Figure 4A). In stark contrast, following CB-5083 or NMS-873 treatment, the abundance of CMs was dramatically decreased with no CM detected at all in 33 CB-5083-treated analyzed cells (Figure 4B). While the few CMs detected in NMS-873-treated cells did exhibit sizes in the range of those in control cells (Figure 4C), their appearance was different with a relaxed electron-dense morphology (Figure 4A, top-right panel). Upon either drug treatments, we noticed the accumulation of vesicles with irregular shapes that might result from CM destabilization (Figure 4A, bottom-right and middle panels). These phenotypes were not attributed to changes in viral protein stability since such short treatment did not impact overall levels of NS4B and NS3, the two components of the DENV CMs (Figure 4D) [8,9]. We and others have previously shown that DENV NS4B and NS3 interact in infected cells [17,18,52]. Considering this, we assessed whether the observed CM alteration correlates with an impairment of NS3/NS4B complexes. We performed co-immunoprecipitation assays with cells co-expressing NS2B/NS3 protease and NS4A-NS4B-HA (Supplementary Materials Figure S2). As previously reported [17], NS3/NS4B-HA interaction was specifically detected. As control, it was completely abrogated when we expressed a NS4B-HA mutant harboring the Q134A mutation in its cytosolic loop [17]. In contrast, neither the CB-5083 nor the NMS-873 four-hour treatment significantly impacted the extent of NS3/NS4B interaction. This strongly suggests that the alteration of CMs upon drug treatment is not due to a loss of NS3/NS4B interaction.

### 4.4. VCP Inhibition Impedes the Biogenesis of the DENV VPs

**Figure 5:**
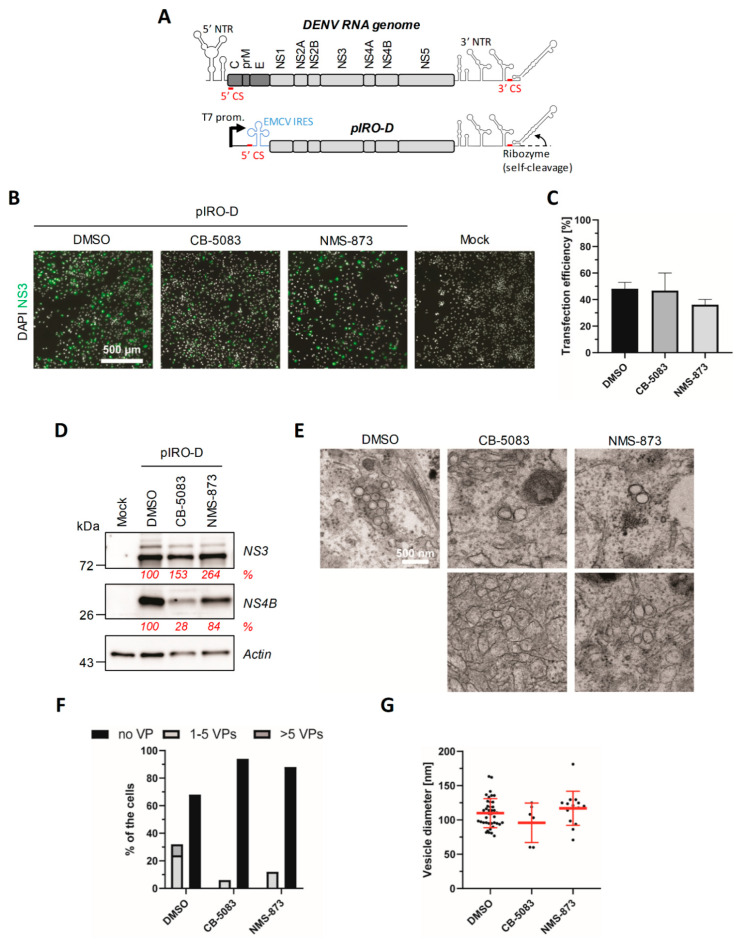
The DENV vesicle packet biogenesis requires VCP ATPase activity. (**A**) Schematic representation of the plasmid-induced DENV replication organelle system (pIRO-D) with a side-by-side comparison between the transcribed RNA with the DENV RNA genome. NTR: non-translated region; prom.: promoter; 5′ CS: 5′ cyclization sequence; IRES: internal ribosme entry site. (**B**–**D**) Huh7**-**Lunet-T7 cells were transfected with pIRO-D plasmid and, 4 h post-transfection, treated with DMSO, 2.5 µM CB-5083, or 1 µM NMS-873 for 12 h. (**B**) Fixed cells were labeled with anti-NS3 antibodies and imaged by widefield microscopy. (**C**) The transfection efficiency upon treatment with the different compounds was determined based on the % of NS3-positive cells. Mean and SD from two experiments are shown. (**D**) Total lysates of transfected and treated cells were analyzed by Western blotting using the indicated antibodies. The relative abundance of NS3 and NS4B (shown in red) was quantified after normalization to actin levels using the Fiji software. (**E**) Electron microscopy analysis of samples treated as in (**B**). (**F**) Quantification of the number of cells exhibiting the VPs. At least 15 cells from 2 different experiments have been analyzed for each condition. (**G**) The diameter of VPs was determined.

In contrast to control cells, the VPs were completely undetectable in DENV-infected cells that were treated for 4 h with CB-5083 or NMS-873 (Figure 4A). This suggests that VCP ATPase activity is required for VP morphogenesis and/or stability. However, given that VCP inhibition impairs DENV replication, we could not rule out that this loss of the VPs was simply due to a potential shutdown of RNA replication (and, hence, viral protein synthesis) rather than a direct effect on VP morphogenesis. Considering this “chicken and egg” situation, an infection-based approach with longer drug treatments was not appropriate to challenge the hypothesis that VCP is involved in VP biogenesis. To tackle this and clearly evaluate whether VCP ATPase activity is required for *de novo* VP formation, we took advantage of a plasmid-induced DENV replication organelle formation system (pIRO-D) reported by us recently [44,45]. This system allows the expression of the DENV NS1-5 polyprotein under the transcriptional control of the T7 RNA polymerase promoter in a replication-independent context (Figure 5A). Following the plasmid transfection of Huh7-derived Lunet cells that stably express the T7 RNA polymerase (Lunet-T7) to allow cytoplasmic transcription, the IRES-driven synthesis of the polyprotein along with the presence of vRNA 5′ CS and 3′ non-translated region, as well as a ribozyme at the 3′ end of the RNA, is sufficient to induce the VPs with an authentic architecture in the absence of viral genome replication. Thus, this system allowed us to study the impact of VCP inhibitor treatments in VP morphogenesis. Lunet-T7 cells were transfected with the pIRO-D DNA construct and treated 4 h later with 2.5 µM CB-5083 or 1 µM NMS-873. Sixteen hours post-transfection, the cells were prepared for widefield and electron microscopy. Either drug treatment did not have a major impact on the transfection efficiency since the percentage of NS3-positive cells was comparable in all conditions (Figure 5B,C). Moreover, Western blot analysis of cell extracts showed that NS3 expression levels remained unchanged upon treatment (Figure 5D), supporting that VCP inhibition does not impact overall polyprotein synthesis. Interestingly, NS4B levels were decreased by both VCP inhibitor treatments suggesting that VCP ATPase activity is required for NS4B expression at the post-translational level through their interaction (Figure 5D). Very strikingly, transmission electron microscopy analysis of pIRO-D-transfected cells revealed that, despite unchanged polyprotein abundance, only 5–10% of drug-treated cells contained VPs as compared to 30% positive cells in the DMSO control condition (Figure 5E,F). Moreover, in that subset of treated cells, we never detected more than two spherules per VP (Figure 5E, top middle and right panels). Notably, very few were observed in the CB-5083 condition (6 VPs vs. ˃ 40 VPs in DMSO-treated cells for 15 cells analyzed). Most importantly, in NMS-873-treated cells, VPs often exhibited an aberrant morphology in stark contrast with their usual circular shape observed in the DMSO control (Figure 5E, bottom right panel). However, the size of VPs in drug-treated cells remained unchanged (Figure 5G). Furthermore, the CB-5083 treatment also resulted in the aggregation of vesicular structures with irregular shapes in some cells (Figure 5E, bottom middle panel), but these structures were not classified as VPs in our quantification given their distinct morphology. Overall, these results unambiguously demonstrate that proper ER remodeling during VP biogenesis is dependent on the ATPase activity of VCP.

## 5. Discussion

Using both pharmacological and dominant-negative mutant overexpression approaches, we show in this study that VCP, through its ATPase activity, is required for DENV replication in cell culture. This is consistent with an important role of VCP in the homeostasis of the ER, an organelle that is morphologically altered by the DENV to generate vROs. Such viral dependency on this host factor was not surprising, since it is reminiscent to what was reported by us and others for several flaviviruses, such as the ZIKV, WNV, YFV, and JEV [11,39,40,41,42,43]. More specifically, similarly to the DENV, we had previously reported that VCP associates with ZIKV NS4B and that short inhibition of its ATPase activity destabilizes CMs in infected cells [11]. More recently, Sehrawat and colleagues showed that VCP is associated with JEV replication complexes [43]. An interaction with NS5 was detected in infected cells, but not when this viral protein was expressed alone. This is consistent with the idea that NS4B might recruit VCP to vROs. While we were preparing this manuscript, another study identified VCP as a protein partner of both JEV NS4B and DENV NS4B in overexpressing cells [41], confirming our results. Overall, this suggests that flaviviruses share conserved VCP co-opting mechanisms during intracellular viral replication. 

Very importantly, neither VPs nor CMs were detected in infected cells following a 4-h treatment with VCP inhibitor CB-5083. In DENV-infected cells, a fraction of VCP did accumulate to large NS4B-containing punctae. Using CLEM, we had previously demonstrated that these dsRNA-free ultrastructures are CMs [9]. Moreover, a 4 h treatment with NMS-873 or CB-5083 induced a dramatic loss of DENV CMs while overall levels of both NS4B and NS3, two components of CMs, remained unchanged. Such rapid destabilization of CMs is consistent with the fact that CMs are very dynamic structures in terms of size, as previously observed in live-cell imaging of infected cells [9]. Overall, this supports a model in which VCP is physically recruited to CMs through its interaction with NS4B and regulates their stability and/or biogenesis. The exact role of CMs during flavivirus replication remains poorly understood. Since NS3 protease is enriched in this structure, it was for long believed that it is the site of polyprotein maturation. However, this process occurs post-translationally and CMs appear composed of smooth ER and devoid of ribosomes in electron microscopy. Being sometimes located in the same ER network as VPs [8], CMs might be required for VP biogenesis, or vice-versa. In addition, we favor a model in which CMs indirectly support intracellular replication by modulating cellular processes that are detrimental to the efficacy of the viral life cycle. For instance, we have recently shown that VCP ATPase inhibition-mediated CM destabilization stimulates ZIKV-induced apoptosis, supporting that CMs delay cytopathic effects potentially to maximize viral replication at the late time point of infection [11]. Moreover, this inhibition was associated with a loss of the elongated morphology of mitochondria, which are normally located in the vicinity of CMs. In the case of the DENV, mitochondria elongation was shown to promote viral replication while dampening early innate immune responses by decreasing RIG-I translocation to mitochondria-associated membranes [9]. Overall, this supports that flaviviral CMs contribute to replication by attenuating antiviral cellular responses. Very interestingly, Tabata and colleagues have recently reported that VCP inhibition by another drug, DBeQ, increased the half-life of JEV NS4B and increased the abundance of this viral protein in JEV CMs [41]. This is in contrast with what we observed for DENV NS4B in this study. Indeed, a 12 h treatment with VCP inhibitors reduced the levels of NS4B in a replication-independent DENV polyprotein expression set-up (Figure 5D). Such phenotype was not observed for NS3, supporting that VCP controls the stability of NS4B. Nevertheless, a shorter treatment destabilized CMs in infected cells without affecting NS4B abundance (Figure 4D). One possibility explaining the decrease in NS4B levels in longer treatments could be that the integrity of CMs is required for the stability of the NS4B molecules residing in these ultrastructures. This potential reciprocal functional relationship between NS4B and CMs as well as the apparent differences between DENV and JEV NS4B will have to be addressed in future studies. Regardless, this also raises the hypothesis that VCP also locally controls the proteostasis of specific host factors potentially “trapped” in CMs to modulate cellular pathways and promote viral replication. 

It is noteworthy that, in contrast to the VPs, CMs were not detected in DENV-infected C6/36 mosquito cells when analyzed by electron microscopy [7], and VCP insect ortholog TER94 is required for the early steps of the ZIKV life cycle in AF5 mosquito cells [42]. While we cannot completely exclude that CMs are generated in the infected insect, this might reflect the difference between insect and mammals regarding virus-induced cellular responses, such as cytopathic effects and/or innate immunity. For instance, there are no RIG-I-like receptors in insects that instead, primarily use the Toll, Imd, and RNA interference pathways as innate immune systems [53]. Considering this, the reported CM functional interaction with the RIG-I pathway in human cells would be irrelevant and useless in insects.

By taking advantage of a novel replication-independent vRO-induction system, we further demonstrate in this study that VCP ATPase activity is required for VP biogenesis. Considering that DENV NS4B (1) is absolutely required for RNA replication [17,18,19]; (2) localizes to VPs as observed by transmission electron microscopy following immunogold labelling [8,9]; and (3) associates with VCP (this study and [41]), it is tantalizing to speculate that VCP directly regulates VP morphogenesis through its binding to NS4B. Future studies will determine whether VCP ATPase inhibition disrupts interactions between NS4B (or the NS4A-NS4B precursor) with other DENV proteins, which are believed to be important for vRNA replication and/or VP biogenesis. While our results show that the stability of the DENV NS4B/NS3 complex is not disrupted by a 4 h treatment with VCP inhibitors, VCP might regulate DENV NS4B *de novo* interactions with NS1 or NS4A [54,55]. Alternatively, one cannot exclude that VCP is required for VP morphogenesis because of its contribution to ER homeostasis, notably to the ER-associated degradation (ERAD) by retrotranslocating misfolded proteins from this organelle and targeting them to the proteasome [29,30,33]. It was recently reported that JEV replication is decreased upon knockdown of VCP co-factor UFD1, which confers substrate selectivity to the complex for proteasomal targeting [43]. Consistently, UFD1 was also identified as a protein partner of both JEV-NS4B and DENV NS4B [41]. However, JEV replication did not require the co-factor UBXD1 [43], suggesting some selectivity regarding the flaviviral co-opting mechanism of VCP complexes and potentially the substrates targeted for degradation. It will be relevant to investigate in future studies whether DENV infection modulates VCP substrate selectivity and its interactome in terms of its 30 co-factors identified so far. More generally, it is tempting to hypothesize that VCP regulates the flavivirus life cycle in association with other known NS4B protein partners. For instance, transmembrane protein 41B (TMEM41B) was identified as a ZIKV NS4B protein partner, which is required for viral replication [56]. More recently, Hoffmann and colleagues showed that this protein is a pan-flaviviral host dependency factor [57]. Both studies showed that TMEM41B redistributed to NS4B-containing large punctae, similarly to VCP. Interestingly, while YFV replicated much less efficiently in TMEM41B-knock-out cells, interferon-stimulated genes and apoptosis were more induced than in wild-type cells. As an apparent ER resident protein, it was proposed that TMEM41B contributes to ER membrane remodeling during vRO biogenesis. All these data are relevant to the proposed role of VCP in this latter process (this study), as well as to the modulation of early innate immunity and cytopathic effects by CMs [9,11]. Thus, it is plausible that TMEM41B and VCP regulate the flavivirus life cycle within the same complex. Alternatively, VCP might also contribute to the NS4B-mediated downregulation of stress-associated ER protein 1 (SERP1), a reported DENV2 restriction factor [58].

Finally, it is noteworthy that several studies have also reported an additional role of VCP during the post-fusion steps of the YFV and ZIKV life cycles in both human and mosquito cells [40,42]. This highlights that VCP impacts the flavivirus life cycle at multiple levels and, hence, VCP-targeting inhibitors are expected to possess a high antiviral potency *in vivo*. In a murine JEV-infection model, administration of CB-5083 in infected mice reduces the viral load in the brain and significantly delays the mortality rate [43]. Unfortunately, the clinical trials assessing the anti-cancer activity of CB-5083 were stopped because of patients experiencing visual loss [59,60]. A more selective and bioavailable analog, CB-5339 is currently being challenged in phase I clinical trials for treatment of acute myeloid leukemia and myelodysplastic syndrome, as well as solid tumors and lymphomas (www.clinicaltrials.gov; identifiers NCT04372641 and NCT04402541) (accessed on 17 July 2021). If this second generation oral drug is safe in humans, it will be highly relevant to assess whether it can be repurposed for the treatment of infection with the DENV and, ideally, as a broad-spectrum antiflaviviral drug. 

## Data Availability

Not applicable.

## Supplementary Materials

The following are available online at https://www.mdpi.com/article/10.3390/v13102092/s1, Figure S1: VCP ATPase inhibition impairs the replication of DENV strains from all four serotypes; Figure S2: VCP ATPase inhibition does not impair NS4B/NS3 interaction.
