# Supplementary material for: The Biogenesis of Dengue Virus Replication Organelles Requires the ATPase Activity of Valosin-Containing Protein
Source: Viruses. 2021 Oct 18;13(10):2092. doi: 10.3390/v13102092 (PMC8540793; doi:10.3390/v13102092)
Supplement: Supplementary file 1 [file viruses-13-02092-s001.zip › viruses-1426677-supplementary.pdf]

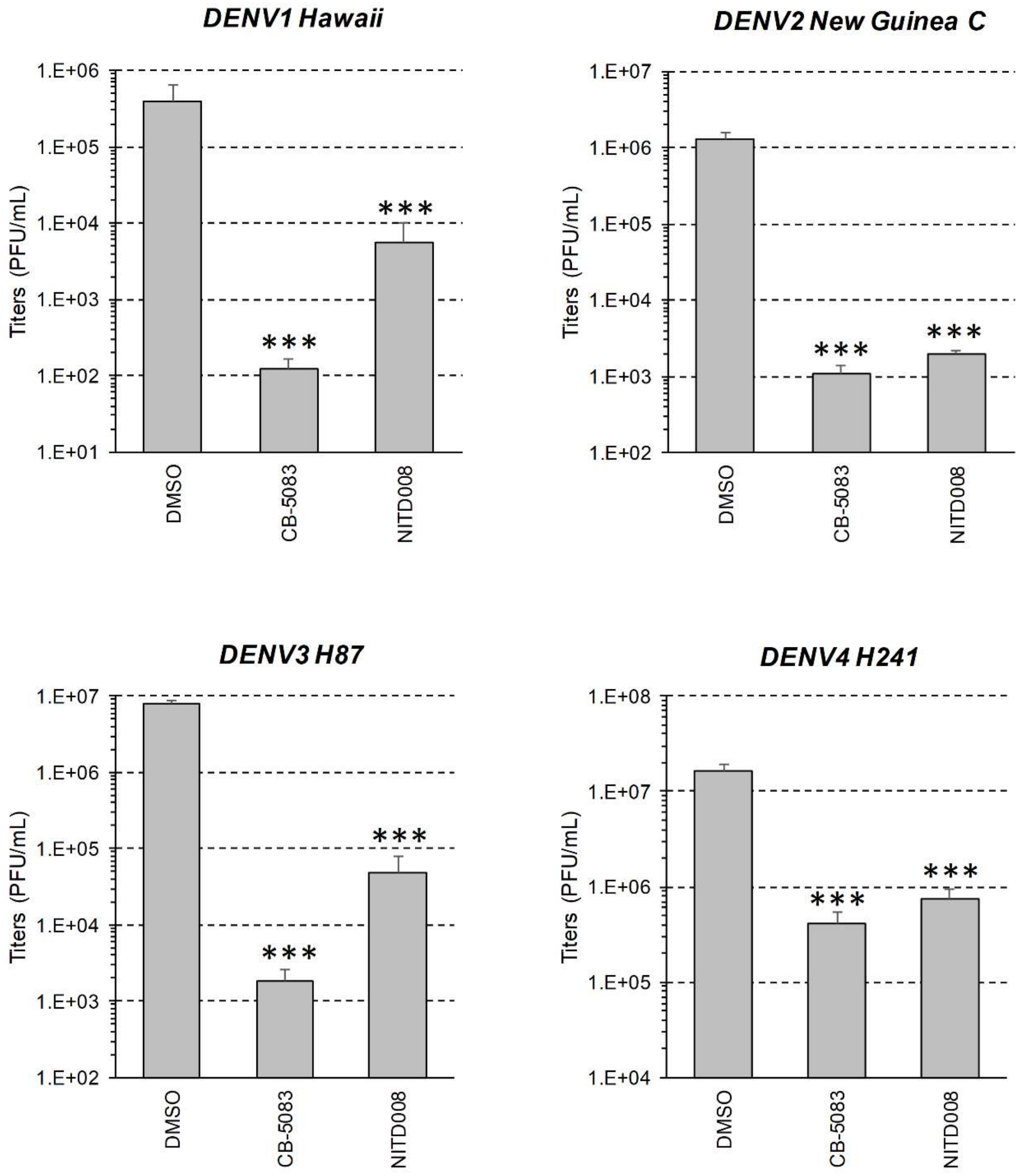

Figure S1 : VCP ATPase inhibition impairs the replication of DENV strains from all four serotypes. Huh7.5 cells were infected with the serotype 1 DENV1 Hawaii strain, the serotype 2 DENV2 New Guinea C strain, the serotype 3 DENV3 H87 strain or the serotype 4 DENV4 H241 strain at a MOI of 0.1-0.05. The day after, cells were treated with DMSO, 0.5 μM CB-5083 or 10 μM NITD008 as negative control. 24 hours later (2 days post-infection), cell supernatants were collected and plaque assays were performed. ***: p-value ≤ 0.001.

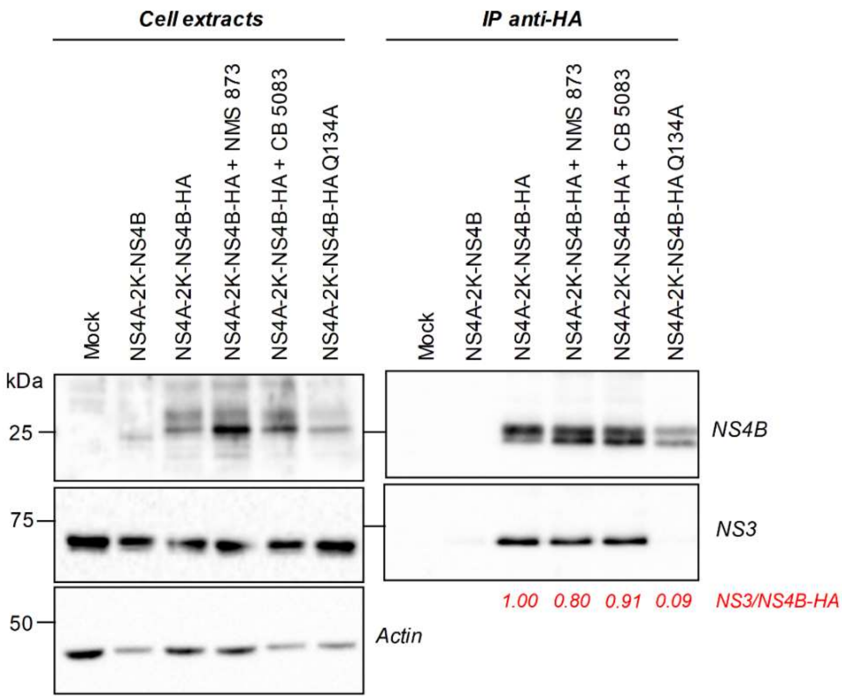

Figure S2 : VCP ATPase inhibition does not impair NS4B/NS3 interaction. Huh7.5-T7/NS2B3 cells were transfected with the plasmids expressing the indicated viral proteins. 12 hours post-transfection, cells were treated with DMSO, 10 µM NMS-873 or 10 µM CB-5083. Four hours later, cells extracts were prepared and subjected to co-immunoprecipitation directed against HA. Resulting eluates and cell extracts were analyzed by western blotting using the indicated antibodies. The NS4A-2K-NS4B-HA Q134 mutant was used as a positive control since this mutant was reported to be unable to associate with NS3 [17]. The relative abundance of NS3 in the IP (shown in red) was quantified after normalization to pulled-down NS4B levels using the ImageLab software (Bio-Rad).
